# Reticulocalbin-1 in clear cell renal cell carcinoma: clinical and functional evidence for its role as a biomarker and potential therapeutic target

**DOI:** 10.1186/s12885-025-14817-2

**Published:** 2025-09-20

**Authors:** Finn Krause, Michael Stoffel, Franziska I. Winterhagen, Jörg Ellinger, Glen Kristiansen, Manuel Ritter, Marieta Toma

**Affiliations:** 1 Institute of Pathology, University Hospital Bonn, 53127 Bonn, Germany; 2 Department of Urology, University Hospital Bonn, 53127 Bonn, Germany

**Keywords:** Clear cell renal cell carcinoma, *RCN1*, Biomarker, Target-identification, Survival

## Abstract

**Background:**

Clear-cell renal cell carcinoma (ccRCC) is the most prevalent subtype of renal cell carcinoma, and its prognosis in a metastatic stage is poor. Although therapeutic options are continuously improving, better combination therapies and individualized approaches are still needed. Reticulocalbin-1 (*RCN1*), located in the endoplasmic reticulum (ER), is associated with aggressiveness and poor prognosis in many solid tumors, but its role in ccRCC has not been analyzed before.

**Methods:**

In this study, we performed in-silico transcriptomic data mining to analyze *RCN1* expression at mRNA and protein levels using large publicly available databases and conducted the first large-scale cohort study on the impact of *RCN1* in ccRCC, including data from 306 patients who underwent tumor resection at the Clinic of Urology, University Hospital Bonn. We examined the correlation of *RCN1* expression with clinicopathological parameters and overall survival. Additionally, we analyzed the association of *RCN1* expression with CD8 T-lymphocyte and macrophage infiltration. In vitro functional analysis was performed by silencing *RCN1* using siRNA in Caki-1 and A498 cell lines to determine its role in tumor cell behavior.

**Results:**

*RCN1* is highly expressed in ccRCC at both the mRNA and protein levels in public databases, which was confirmed by our cohort data, where *RCN1* was found to be highly and homogenously expressed in 63.7% of ccRCCs. High *RCN1* expression was associated with shorter overall survival both at the mRNA (*p* < 0.001) and protein levels (*p* = 0.01). Furthermore, high *RCN1* expression was correlated with higher tumor grade (*p* = 0.002), tumor stage (*p* = 0.036), presence of lymph node metastases (*p* = 0.004), and distant metastases (*p* = 0.017). Clusters of macrophages tended to correlate with *RCN1* expression (*p* = 0.051), but no significant correlation was found between *RCN1* expression and the amount of CD8 T-lymphocytes. Additionally, silencing *RCN1* led to a significant reduction in tumor cell migration and invasion.

**Conclusion:**

Our results confirm that *RCN1* is highly and homogenously expressed in ccRCC and correlates with poor prognosis and unfavorable clinicopathological parameters. *RCN1* could serve as a reliable biomarker for prognosis in ccRCC and shows potential as a target for therapeutic approaches.

## Introduction

Kidney cancer is the third most common malignant tumor in urology, with a worldwide incidence of 431,300 and 179,400 deaths in 2020 (https://gco.iarc.fr/today/en). About 90% of kidney tumors are renal cell carcinomas (RCC), the most common subtype, with around 75% of all cases, being the clear cell renal cell carcinoma (ccRCC) [1].

A significant challenge in ccRCC disease management is its propensity for metastasis; at initial diagnosis, around 20–30% of patients present with metastatic disease, and nearly 30–50% of patients who undergo nephrectomy for localized disease eventually develop metastases [2, 3].

Treatment for metastatic ccRCC has evolved significantly over the past decade, shifting from cytokine-based therapy to targeted molecular therapies and immune checkpoint inhibitors. Current first-line treatment options include tyrosine kinase inhibitors (TKIs) such as sunitinib and cabozantinib, immune checkpoint inhibitors (ICIs) targeting PD-1/PD-L1 and CTLA-4 (e.g., nivolumab, pembrolizumab, and ipilimumab), and combination regimens involving both TKIs and ICIs [4].

In recent years, survival time has improved as new therapeutic approaches have been continuously developed. While there are some prognostic markers available, including clinical and laboratory parameters [5, 6], there have been many attempts to find and establish new markers with better prognostic value, some of which have been suggested as therapeutic targets as targets of an antibody–drug-conjugate [7].

The protein reticulocalbin-1, which is encoded by the *RCN1* gene, is a member of the CREC family. A protein family with a conserved Ca^2+^ -binding site, the EF-Hand motif. Reticulocalbin-1 is localized in the ER-lumen and has a low binding affinity to Ca^2+^ [8]. Its expression was observed in various non-malignant tissues, particularly in glands, suggesting a potential role in biosynthetic or secretory pathways [9]. Notably, the conformation of reticulocalbin-1 changes depending on the number of bound calcium ions and responds to small changes of Ca^2+^ concentration in the ER. This observation has led to the hypothesis that *RCN1* may be involved in calcium-dependent secretory pathways [10]. While its function in normal tissues is poorly understood, *RCN1* upregulation has been associated with various tumors, e.g. glioblastoma, lung carcinomas, prostate adenocarcinomas and others [11–15].

One established function of *RCN1* is the inhibition of ER stress-induced apoptosis [16]. This effect was discovered in RCC and hepatocellular carcinoma (HCC) cell lines and is suggested to be the cause of therapy resistance against the anthracycline Adriamycin in nasopharyngeal carcinoma and the tyrosine-kinase-inhibitor sorafenib in HCC [17, 18]. Fu et al. further suggested that reducing ER stress also enhances the cells' viability to migrate [19].

Nevertheless, very few data for *RCN1* regarding renal cell carcinoma are available. In 2013, a proteomics-analysis-based study was published, which suggests a potential role of *RCN1* as a biomarker in RCC. In this study, reticulocalbin-1 was significantly upregulated in all 24 examined cancer tissue samples in comparison to non-neoplastic kidney tissue. While there was no significant correlation between *RCN1* expression and TNM stage, the authors acknowledge that due to their limited cohort of 24 cases, the power of this analysis is low [20]. About ten years later, the influence of tumor-infiltrating regulatory T cells on the prognosis of ccRCC was analyzed and a correlation between the overall survival, Treg-cell infiltration and *RCN1* was determined. The authors propose *RCN1* as a potential ccRCC biomarker [21].

To our knowledge, this is the first study on a large cohort of ccRCC patients inquiring the expression of *RCN1* concerning clinicopathological parameters and survival. Moreover, we employed in vitro experiments to decipher the functional aspects of *RCN1* in ccRCC. This study aims to investigate the *RCN1* expression in clear cell renal cell carcinoma in correlation to different stages, tumor grades and overall patient survival and link the *RCN1* expression to immune cell infiltration in ccRCC. We conducted in vitro experiments downregulating *RCN1* in ccRCC cell lines to elucidate the potential role of *RCN1* in tumor aggressiveness in ccRCC.

## Materials and methods

### Patients and samples

The cohort consists of 306 patients with ccRCC who underwent radical or partial nephrectomy between 2006 and 2017 at the University Hospital Bonn. The studies were approved by the Ethics Committee of the University Hospital Bonn (EK 233/20). The clinicopathological characteristics of the patients are summarized in Table 1.

**Table 1:**
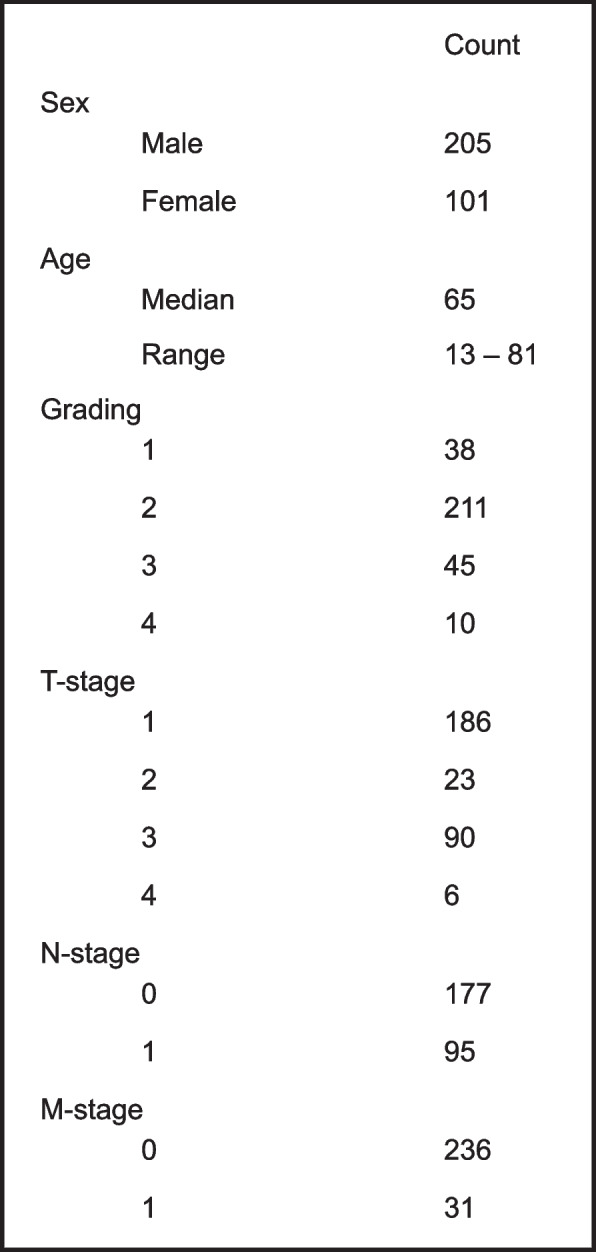
Description of our TMA cohort and its characteristics: sex, age, grading, TNM-stage

### Tissue microarrays and immunohistochemistry for reticulocalbin-1

Tissue microarrays (TMA) were manually constructed. Representative tumor and non-neoplastic areas were selected on hematoxylin and eosin staining and punched for TMA blocks. Immunohistochemistry (IHC) was carried out to standardized protocols. In short, formalin-fixed, paraffin-embedded tissue from renal carcinomas and non-neoplastic tissue were used to construct TMA. Per case, two tumors and two normal tissue spots (diameter 1 mm) from every patient were punched. A 2-µm section from each TMA was cut and mounted on superfrost slides (Fa. Menzel Gläser, Brunswick, Germany). After deparaffinization with xylene and gradual rehydration, antigen retrieval was performed. The slides were stained with an antibody against reticulocalbin-1 (Fa. Invitrogen, Clone PA5-64,004; dilution 1:50) on the autostainer BenchMark Ultra (Fa. Roche) followed by UltraView detection (Fa. Roche). We used hematoxylin for the counterstaining.

**Fig. 1:**
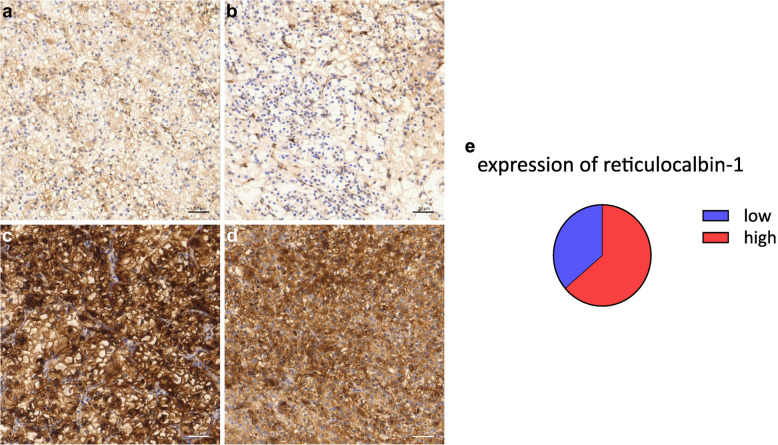
**a**-**d** reticulocalbin-1-staining of our TMA-cohort, categorization in groups of high or low respectively no expression of reticulocalbin-1: **a** low, **b** low, **c** high, **d** high. **e** distribution in high and low/no expression of reticulocalbin-1; red: 64 % high expression, blue: 36 % low/no expression

The immunohistochemical staining was evaluated under the microscope, blind to clinical outcome, clinical and pathological stage. Staining intensities were graded for the cytoplasm of tumor cells or non-neoplastic tubulus cells. A 4-tier grading system (0: negative; 1: weakly positive; 2: moderately positive; 3: strongly positive) was used. We decided not to use the H-score since homogenous staining in each core was observed. For further statistical analyses, we defined a staining intensity ≤ 1 as a low level and a staining intensity > 1 as a high level of staining (Fig. 1a-d).

### Correlation analysis between *RCN1* mRNA expression, molecular-pathological characteristics and survival

We analyzed and visualized the mRNA and protein level of *RCN1* using the UALCAN interface and the publicly available protein (CPTAC) and mRNA (TCGA) databases [22, 23]. We confirmed the analysis using cBioPortal for cancer genomics (https://www.cbioportal.org). For survival analysis, we downloaded the normalized mRNA values from oncolnc (www.oncolnc.org) and split the groups into low and high expression, separated at the median expression.

### Cell culture and transfection

We used the RCC cell lines Caki-1 (ATCC HTB-46) and A498 (ATCC HTB-44) for all experiments. The cell lines were cultured in McCoy medium (Caki-1) or DMEM + GlutaMax medium (A498) containing 10 % fetal calf serum (FCS), 1 % PenStrep and were maintained in a humidified atmosphere with 5 % CO2 at 37 °C. To achieve a transient knockdown of the activity of reticulocalbin-1, we transfected the cells with siRNA targeted against *RCN1*-mRNA (Fa. Qiagen). The sequences used for knockdown of *RCN1* expression were siRCN1#1, ATCTTTGATAATGTCGCCAAA; siRCN1#2, ATGAGCTTTGATAGACACTCA; siRCN1#3, CTGGATCCTCCCTCAAGATTA; siRCN1#4, AAGGACGGGAAGTTAGACAAA.

Cells were seeded at a concentration of 200.000 (Caki-1) or 100.000 (A498) cells/well in a 6-well plate cell medium with 10 % FCS. After 24 h, 5 µL Lipofectamine 3000 (Fa. Invitrogen) was mixed with 3 µL siRNA (10 µM) in 250 µL Opti-Mem serum-free medium and added to each well. After 48 h of incubation, the experiment was stopped. Our control cells with scrambled-siRNA-transfection were used for all experiments and treated equally to the knockdown cells.

#### Transfection effectiveness

After 48 h of transfection, we extracted RNA using the RNeasy Mini Kit and the manufacturer's protocol (Fa. QIAGEN). cDNA was synthesized using Superscript IV (Fa. Invitrogen), followed by qPCR using Cybergreen (Fa. BIO-RAD) on the ViiA 7 (Fa. Applied Biosystems). The *RCN1* expression was downregulated to 10 % for Caki-1 and to 15 % for A498.

### Scratch assay

48 h after transfection, the wells were scratched with a 200 µL pipette tip and further incubated. To monitor the progression, images were taken from defined positions: immediately, after 24 h of further incubation for Caki-1 and after 8 h for A498. Migration potential was measured as growth area per time and compared between knock-downed cells and control cells with scrambled siRNA transfection.

### Invasion assay

We used cell culture inserts with 8 µm pores, coated with matrigel-matrix (Fa. Corning) and a fluorescence-blocking membrane for 24-well plates (Fa. Corning). To differentiate the invasion from migration, only half of the inserts were coated. The other half was used as a reference for cell migration. The matrix was incubated at 37 °C for 2.5 h before use. As chemoattractant medium with 10 % FCS was added to the lower chamber. The transfected cells were suspended in a medium without FCS and added to the upper chamber (for Caki-1, 60.000 cells were placed in the upper chamber, for A498, 30.000 cells were placed). After 24 h for Caki-1 and 8 h for A498 of incubation, representative pictures were taken, the number of cells was counted using the program QuPath and the invasion potential was determined as the ratio of invasive cells per migrative cells and compared with the controls.

### Tissue microarrays and immunohistochemistry for CD8 and CD68

CD8 and CD68 were stained using the same protocol on the Medac autostainer 480S (Fa. Medac, Germany). Briefly, after deparaffinization, the slides were incubated with the primary antibody against CD8 (Fa. DAKO, clone C8/144B, dilution 1:50) or CD68 (Fa. DAKO, clone PG-M1, dilution 1:100) respectively, followed by DAB detection. Counterstaining was performed for 3 min using hematoxylin.

**Fig. 2:**
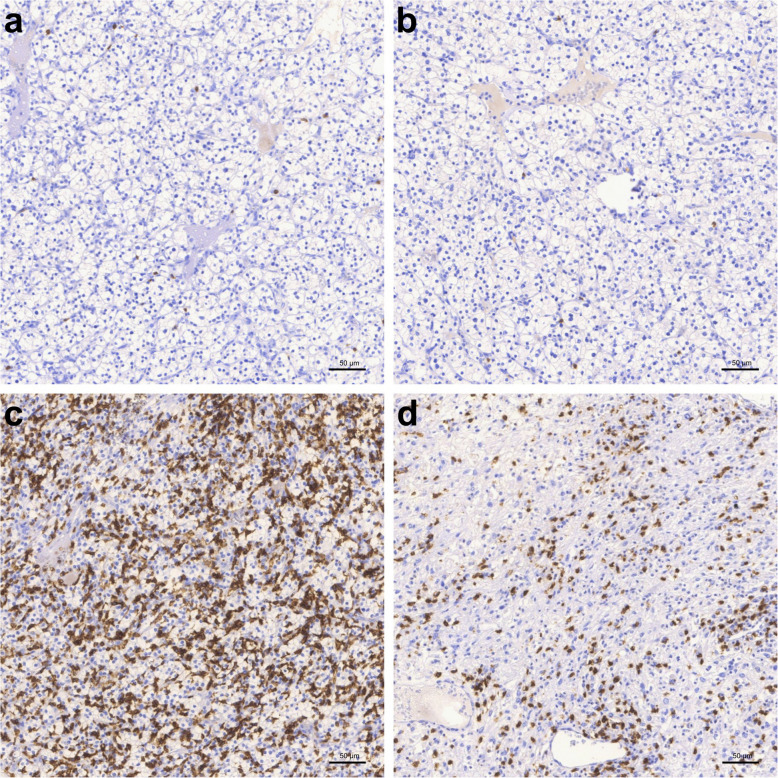
CD8-staining of our TMA-cohort, categorization in groups of high or low respectively no expression of CD8: **a** & **b** low, **c** & **d** high

**Fig. 3:**
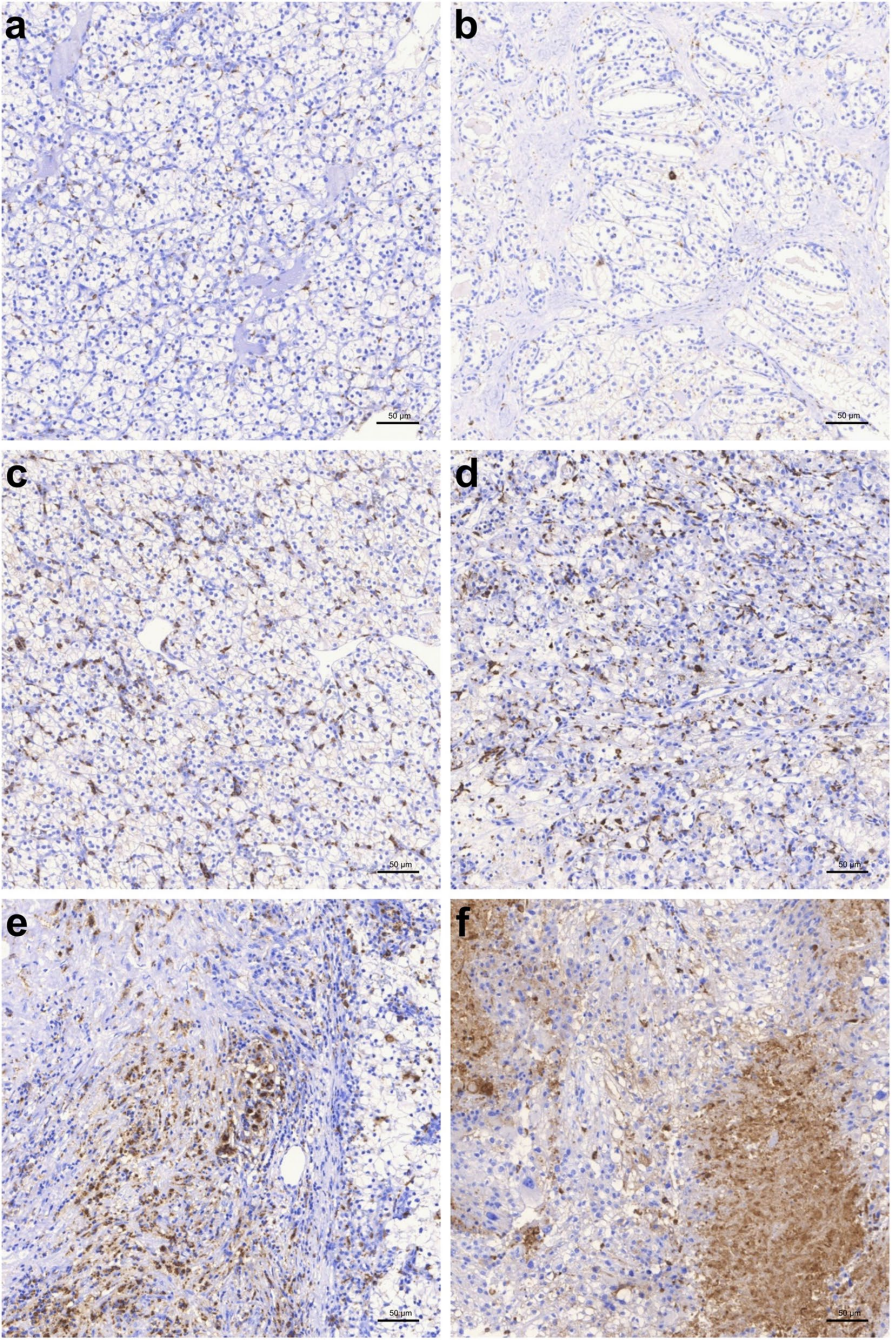
CD68-staining of our TMA-cohort, categorization in groups of no expression, uniform distribution and cluster forming expression: **a** & **b** no expression, **c** & **d** uniform distribution, **e** & **f** cluster forming

Analysis was performed using QuPath and its ability to automatically detect round cells and display the intensity of different light channels. CD8 analysis comprised calculating the number of CD8-positive cells detected by QuPath per mm^3^ of tissue (Fig. 2). For CD68, Chakiryan et al. [24] also described a correlation between a high spatial clustering of CD68-positive cells with worse overall survival in metastatic ccRCC, which is why we also manually divided the CD68 tissue samples into three groups. The first one shows no or very few CD68 positive cells, the second one has a uniform distribution of CD68 positive cells and the last comprises stances where CD68 positive cells form clusters (Fig. 3).

### Data analysis

Data was analyzed using SPSS Version 29 and GraphPrism. The Mann–Whitney test was performed for paired samples. Dunn’s correction for multiple comparisons when performing multiple testing with the Kruskal–Wallis test. For correlation analysis, we used the Pearson correlation test. The Kaplan–Meier plots and the log-rank test were used for survival analysis.

## Results

### Reticulocalbin-1 level is increased in ccRCC compared with non-neoplastic tissue

**Fig. 4:**
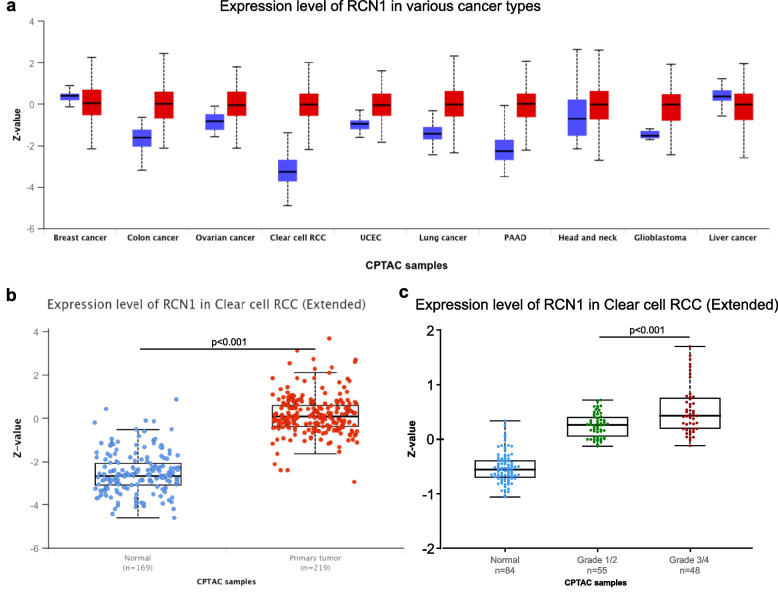
Protein level of reticulocalbin-1 based on data of the CPTAC; **a** comparison between normal tissue (blue bars) and tumor (red bar) across various types of cancer, it is striking that the highest difference is in ccRCC; **b** comparison between normal kidney tissue and ccRCC, ccRCC shows a significant increased expression level of *RCN1*, *p* < 0.001 (Welch’s t-test, analysis by UALCAN); **c** comparison between normal kidney tissue and different gradings in ccRCC, high-grade tumors (G3/4) have a significant increased expression level of *RCN1* compared to low-grade tumors (G1/2), *p* < 0.001 (Mann–Whitney-test)

Using the visualization platform UALCAN [22, 23], we noticed that ccRCC has the highest *RCN1* protein level compared with normal tissue across the tumors as analyzed in CTPAC data set (Fig. 4a. The comparison between non-neoplastic and ccRCC tissue reveals significantly higher *RCN1* levels in tumors (*p* < 0.001; Fig. 4b). *RCN1* levels were significantly higher in high-grade tumors compared with low-grade tumors (G1/2 versus G3/4; *p* < 0.001; Fig. 4c); but no significance regarding gender and race was noticed.

**Fig. 5:**
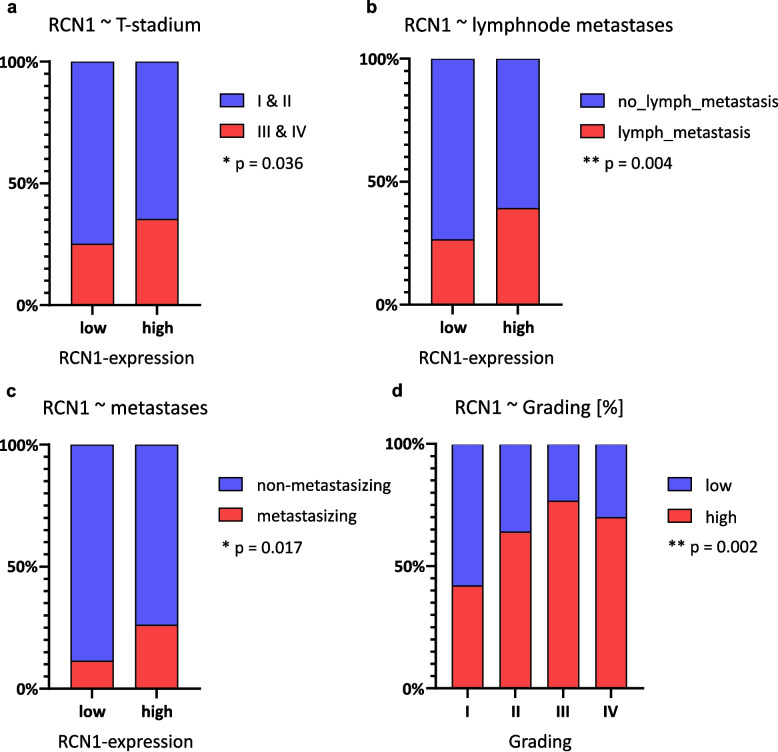
Protein level of reticulocalbin-1 based on data of our TMA; **a** comparison reticulocalbin-1-expression and T-stage, significant correlation between higher reticulocalbin-1-expression and higher t-stage, *p* = 0.036 (Mann–Whitney-Test); **b** comparison reticulocalbin-1-expression and N-stage, significant correlation between higher reticulocalbin-1-expression and prevalence of lymph node metastasis, *p* = 0.004 (Mann–Whitney-Test); **c** comparison reticulocalbin-1-expression and M-stage, significant correlation between higher reticulocalbin-1-expression and prevalence of distant metastasis, *p* = 0.017 (Mann–Whitney-Test); **d** comparison of reticulocalbin-1-expression between different gradings in ccRCC, the amount of high-expressive tumors is increased in higher gradings (G 3/4) compared to lower gradings (G1/2), *p* = 0.002 (Mann–Whitney-Test)

To confirm the results, we analyzed immunohistochemically a large cohort of ccRCC and non-neoplastic kidney tissue. We dichotomized the cohort into high expression and low/no expression and analyzed the *RCN1* level according to the clinicopathological parameters. Out of 306 ccRCC, 195 cases (63.7%) showed a strong *RCN1* level, while 111 tumors (36.3%) were negative or low positive (Fig. 1). We could confirm that the *RCN1* level correlated significantly and positively with tumor grading (*p* = 0.002), pT stage (*p* = 0.036), presence of lymph node metastases (*p* = 0.004) and distant metastases (*p* = 0.017) (Fig. 5).

**Fig. 6:**
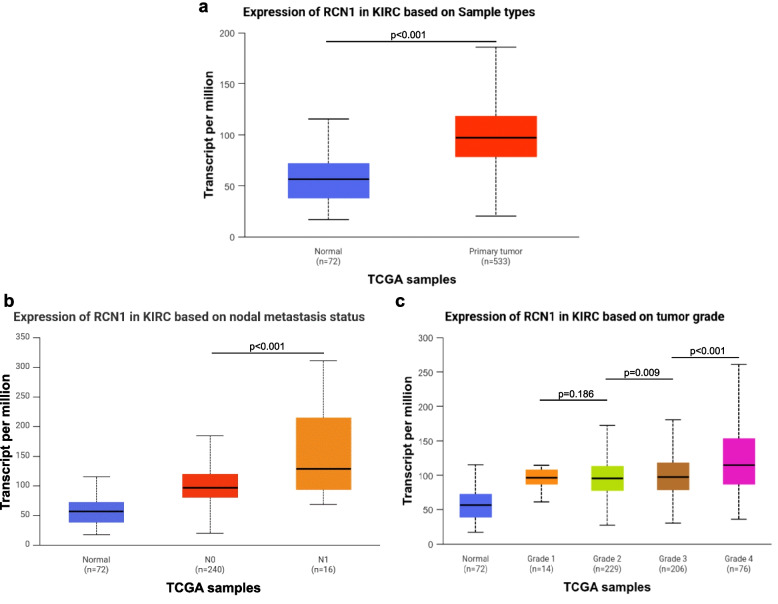
mRNA expression of *RCN1* based on data of TCGA; **a** comparison of mRNA-transcripts between normal kidney tissue (blue) and ccRCC, ccRCC shows an increased level of transcription of *RCN1* into its mRNA, *p* < 0.001 (Welch’s t-test, analysis by UALCAN); **b** comparison of mRNA-transcripts between normal kidney tissue and N-stage in ccRCC, tumors with lymph invasion (N1) have a significant increased expression level of *RCN1* compared to tumors without (N0), *p* < 0.001 (Welch’s t-test, analysis by UALCAN); **c** comparison of mRNA-transcripts between normal kidney tissue and different gradings in ccRCC (Kruskal–Wallis-test, Dunn’s comparison)

Next, we proved whether the *RCN1* mRNA expression correlates with the protein level. Therefore, we used the UALCAN visualization platform on the TCGA data set. Additionally, we downloaded and analyzed the normalized mRNA expression of *RCN1* (www.oncolnc.org) in ccRCC and non-neoplastic tissue. *RCN1*-mRNA was highly expressed in ccRCC compared with non-neoplastic tissues (Fig. 6a). Regarding clinicopathological parameters, tumors with lymph node metastases had a significantly higher *RCN1* mRNA expression compared with tumors without lymph node metastases (*p* < 0.001) (Fig. 6b). In addition, the *RCN1* mRNA expression increased significantly with the tumor grading (Fig. 6c).

### Increased *RCN1* is associated with shorter survival time in ccRCC

**Fig. 7:**
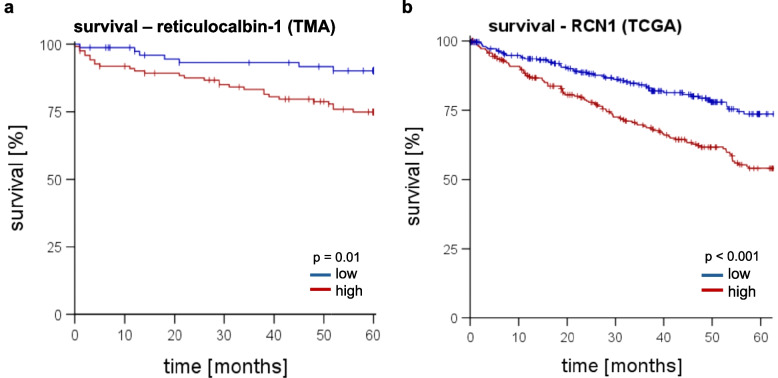
Survival-time-analysis at high (red) and low (blue) expression of reticulocalbin-1; **a** survival-time-analysis based on data of our TMA-cohort, patients with higher expression of reticulocalbin-1 had a significantly shorter survival time, *p* = 0.01 (log-rank test); **b** survival-time-analysis based on data of the TCGA-cohort, patients with higher expression of reticulocalbin-1 had a significantly shorter survival time, *p* < 0.001 (log-rank test)

Further, we analyzed the impact of reticulocalbin-1 on the 5-year patients'survival. After 5 years, significantly more patients with low or no reticulocalbin-1 levels were alive, compared with patients with a high level (log-rank test, *p* = 0.01). After 5 years, only 10 % of patients in the low-expression group died, while around 26 % of the patients in the high-expression group were dead (Fig. 7a). Also, regarding the data of TCGA, the mRNA expression of *RCN1* was correlated with overall survival, patients with *RCN1* expression more than the median had a shorter overall survival than patients with lower *RCN1* expression (log-rank test, *p* < 0.001; Fig. 7b).

### The suppression of reticulocalbin-1 can reduce the migration and invasion potential in ccRCC

**Fig. 8:**
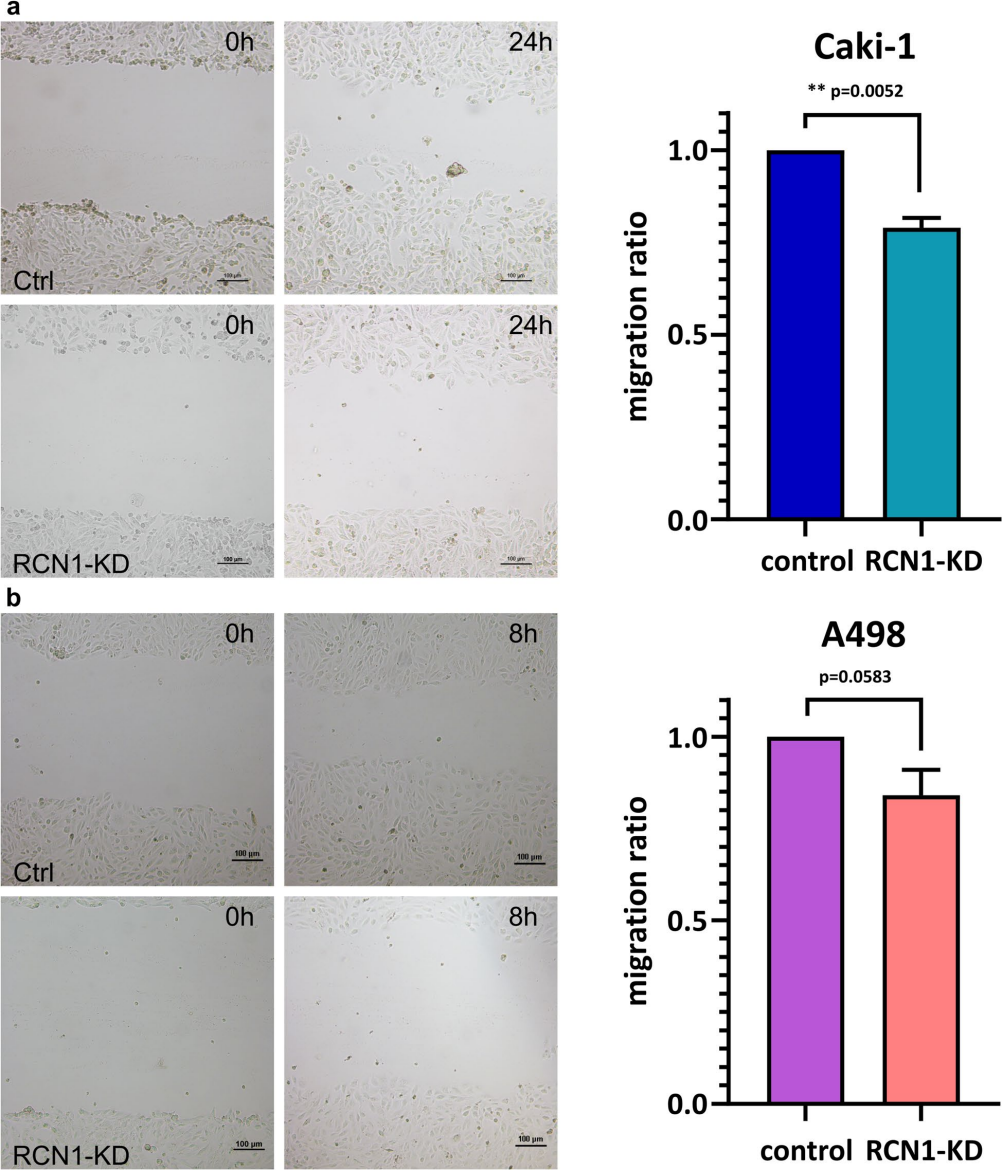
Scratch-assay with *RCN1*-siRNA knockdown-cells (*RCN1*-KD) and scrambled-siRNA control cells (Ctrl); **a** scratch-assay with cell line Caki-1, cells with *RCN1* knockdown show significantly less migrative movement in comparison to the control cells with scrambled siRNA after 24 h, *p* = 0.0052 (Welch’s t-test); **b** scratch-assay with cell line A498, cells with* RCN1* knockdown show no significant difference in migrative movement compared to the control cells with scrambled siRNA after 8 h, *p* = 0.0583 (Welch’s t-test)

To explore the *RCN1* functions in ccRCC, we tested the effect of a knockdown of reticulocalbin-1 on the migration potential using a scratch assay. We could detect a significant correlation between the knockdown and a decrease of the migratory potential in the Caki-1 cell line (*p* = 0.0052) (Fig. 8a), and a tendency in A498 (*p* = 0.0583) (Fig. 8b).

**Fig. 9:**
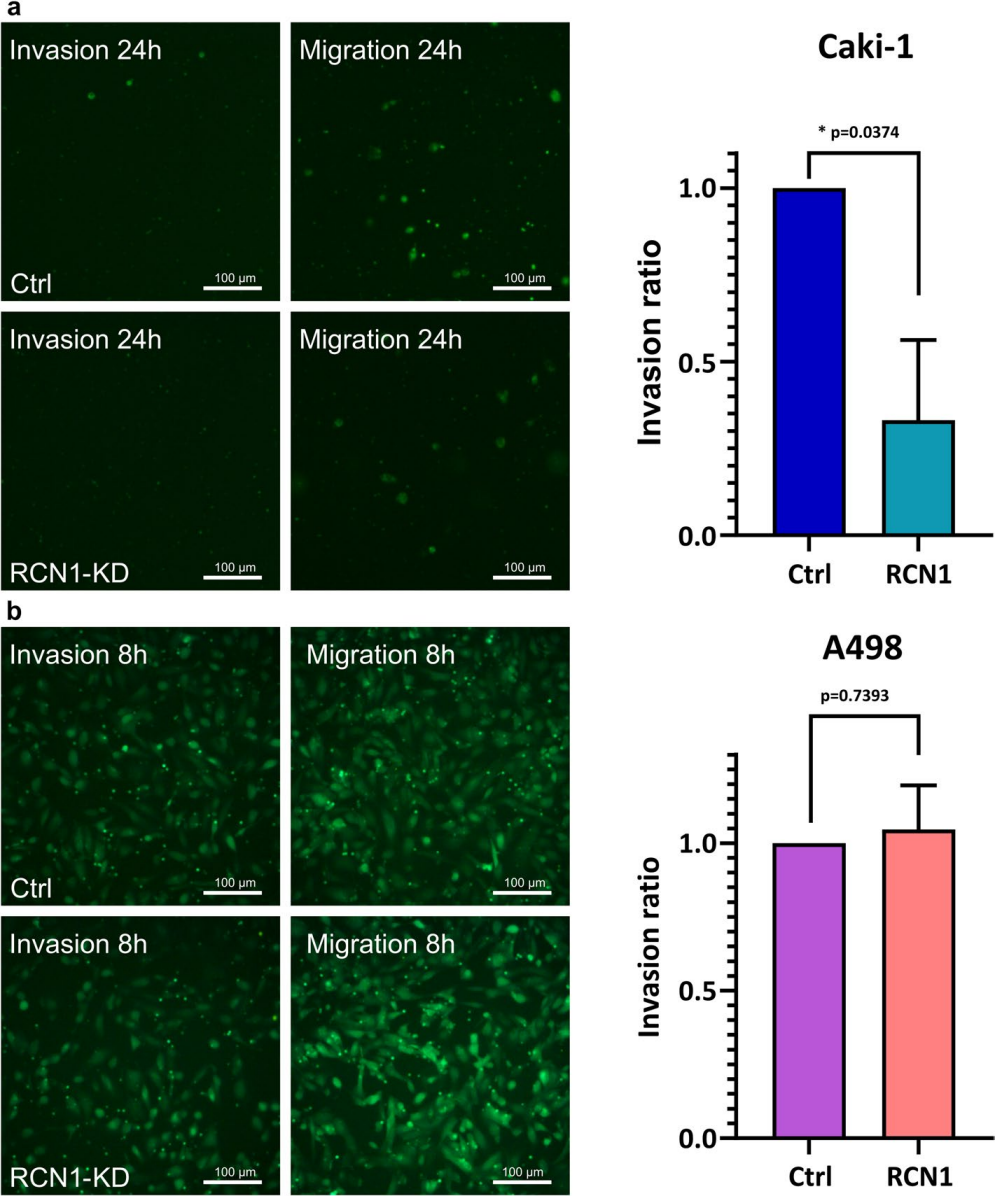
Invasion-assay with *RCN1*-siRNA knockdown-cells (*RCN1*-KD) and scrambled-siRNA control cells (Ctrl) using Cybergreen in a Boyden-chamber-like assay with a fluorescence blocking membrane; 8**a** invasion-assay with cell line Caki-1, cells with* RCN1* knockdown show significantly less migrative movement in comparison to the control cells with scrambled siRNA after 24 h, *p* = 0.0052 (Welch’s t-test); 8**b** scratch-assay with cell line A498, cells with *RCN1* knockdown show no significant difference in migrative movement compared to the control cells with scrambled siRNA after 8 h, *p* = 0.0583 (Welch’s t-test)

To test the effect of our knockdown on the invasion potential, we used a “Boyden-chamber-like assay”. While the Caki-1 wild type showed a low migration potential, the knockdown cells showed no invasion (*p* = 0.0374) (Fig. 9a), but A498 cells showed no difference in the invasion potential after siRNA knockdown (*p* = 0.7393) (Fig. 9b).

### *RCN1* expression and immune cell infiltration

**Fig. 10:**
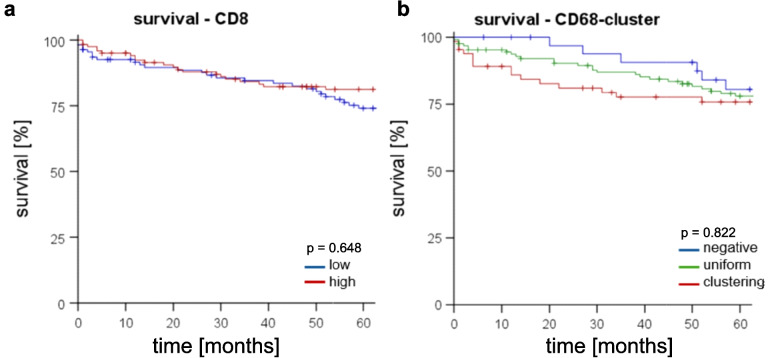
Survival-time-analysis in the context of specific immune infiltration based on data of our TMA-cohort; 7**a** survival-time-analysis in the context of infiltration of cytotoxic T-cells (CD8^+^), no significant difference between high (red) and low (blue) infiltration (log-rank test); 7**b** survival-time-analysis in the context of infiltration of macrophages (CD68^+^), no significant difference between no, uniform and cluster-forming infiltration occurred (log-rank test)

Following a recent paper about the immunological influence on ccRCC published by Qixin et al. [21], we analyzed the amount of cytotoxic T-lymphocytes (CD8-positive) and macrophages (CD68-positive) in ccRCC according to the *RCN1* level (Pearson correlation). No significant correlations between the amount of CD8-positive lymphocytes and the level of *RCN1* were noticed. The presence of macrophage clusters (CD68-positive) had a tendency of significance when correlated to a high *RCN*1 level (*p* = 0.051). The amount of CD8-positive T-lymphocytes correlated significantly with the amount of macrophages and with macrophage cluster formation (*p* < 0.001), but neither the amount of CD8^+^ T-lymphocyte nor CD68-positive macrophage infiltration had a significant impact on patient overall survival in our cohort (Fig. 10).

## Discussion

Despite therapeutic advancements, the prognosis for metastatic ccRCC remains poor, with a 5-year survival rate of approximately 12–20 % [25]. Prognostic models, such as the International Metastatic RCC Database Consortium (IMDC) criteria, help stratify patients based on risk factors, guiding treatment selection and predicting survival outcomes [5]. To improve the outcomes in advanced ccRCC, ongoing clinical trials and basic research continue to focus on biomarker-driven therapies and the discovery of novel targets and treatment combinations.

Our study focuses on *RCN1* and its role in ccRCC, deciphering also the mechanisms involved in cancer progression.

*RCN1* is a Ca2^+^ -binding protein, involved in endoplasmic reticulum stress, highly expressed in several malignant tumors like breast cancer, colorectal cancer, naso-pharyngeal carcinoma or non-small cell lung carcinoma [11, 17, 26, 27] associated either with poor prognosis or therapy resistance. The role of *RCN1* in ccRCC is mainly unknown.

For our investigations, we asked first if *RCN1* could be found at high levels ccRCC. Therefore, we interrogated the CTPAC data set and performed additional immunohistochemistry on a TMA comprising 306 patients. As described in previous publications [11, 17, 26, 27], *RCN1* has a high expression in many solid tumors, but among all solid tumors, ccRCC has the highest *RCN1* level compared with non-neoplastic tissue. We confirmed the results in the immunohistochemistry analysis, the majority of tumors express reticulocalbin-1. This also confirms the proteomics analysis published by Giribaldi et al. [20], who described an *RCN1* overexpression in 21 out of 24 investigated ccRCC specimens.

Alteration of gene expression, either up- or downregulation, does not necessarily correlate with the protein level. Therefore, we analyzed the TCGA data set and demonstrated a high *RCN1* expression in tumors compared with non-neoplastic tissue, in concordance with the protein data.

A high level of *RCN1* was associated with poor clinicopathological parameters, like high grading or high tumor stage. The association between high levels of *RCN1* and prognostic infaust parameters was also described in non-small cell lung carcinoma (NSCLC) and esophageal squamous carcinoma [11, 28].

Overexpression of *RCN1* and high levels of *RCN1* correlate with shorter overall survival in ccRCC, which is in line with the results published for NSCLC and esophageal squamous carcinoma [11, 28]. Experiments in NSCLC and esophageal squamous carcinoma also revealed a significant decrease in invasion and migration after *RCN1* knockdown [11, 28]. In our experiments, the Caki-1 ccRCC cell line showed a significant difference in migration and invasion potential compared to our control. For the A498 cell line (*p* = 0.0583), there is a tendency towards lower migration in the knock-down cell line but no detectable effect on the invasion. A possible explanation for the differences between the cell lines may lie in the biological characteristics of the A498 cells. A498 behaves far more aggressively regarding cell proliferation, migration and invasion. Moreover, A498 is derived from a primary tumor harboring a *VHL* mutation, while Caki-1 is a *VHL* wild type metastatic ccRCC cell line. Due to the *VHL* mutation, A498 cells may compensate for or mask the effects of *RCN1* knockdown, as this mutation leads to constitutive activation of HIF. HIF induces the expression of growth factors that activate the PI3K/AKT pathway – an established driver of cell migration and invasion [29]. Different origins and mutational statuses can lead to distinct biological behaviors.

While we were able to show effects on cell motility using knockdown experiments, the pathomechanistic pathway remains unclear. Although the pathway related to apoptosis inhibition has already been investigated using HEK- and A498-cells [16], the mechanisms behind cell movement influenced by *RCN1* are yet unknown. This limitation will be addressed in a follow-up study.

We also have to acknowledge a possible influence of tumor environment, e.g. the influence of regulatory T-cells on ccRCC and its interaction with *RCN1* [21], which cannot be examined in a cell-line model. In addition to the influence of CD4-positive T-cells, we analyzed a possible influence of cytotoxic T-cells (CD8^+^) and macrophages in the tumor area. From squamous carcinoma (esophageal and oral) is known that the knockdown of *RCN1* inhibits the polarization of M2 macrophages [28, 30]. We have chosen another approach and quantified the macrophages and the clusters of macrophages by immunohistochemistry. The presence of macrophage clusters showed a tendency to high *RCN1* expression (*p* = 0.051), but our cohort did not show any correlation between the macrophage infiltration, *RCN1* expression and clinical outcome. However, in our experiment, we did not differentiate between M1 and M2 macrophages, since CD68 could be a marker for both subtypes. To confirm that the effect described by Guo et al. does not occur in ccRCC, a differentiation between the types of macrophages should be addressed in further studies. Additionally, we couldn’t notice any significant correlation between CD8 T-lymphocyte infiltration and a high *RCN1* level in ccRCC, so we couldn’t confirm the results published by Qixin et al. [21], who discovered an association of *RCN1* with Tregs across malignant tumors, including ccRCC. Our results show no correlation between macrophages and cytotoxic lymphocytes infiltration and *RCN1* levels in ccRCC.

The expression of *RCN1* is very low in normal tissue but often high in ccRCC tumor cells, which predisposes it as a possible therapeutic target. *RCN1* has been reported to induce resistance to sorafenib (TKI-class) by inhibiting ER stress-induced apoptosis in HCC [18]. As the TKI-class is also used as one of the main therapeutic options in ccRCC, a similar mechanism in ccRCC could indicate *RCN1* suppression to be a promising addition to TKI-based therapies. This possibility requires further investigation. Another possible therapeutic approach is to use the high expression of reticulocalbin-1 as a target for an antibody–drug-conjugate, benefiting from the homogenous distribution in all tumor cells. A previous study by Fukuda et al. [9] described the *RCN1* expression across non-malignant and malignant tissues. *RCN1* is highly expressed in few glands especially in the gonads, tissues with terminal differentiation like muscle cells and neurons, and activated fibroblasts in inflammatory tissue as well as in tumor surrounding tissue. Almost no *RCN1* expression was detected in kidney tissue and many endocrine glands, like thyroid gland or hypophysis, and only low expression was found in organs of the gastrointestinal system. These findings suggest that potential adverse effects may primarily affect the gonads and the central nervous system, but because chemotherapeutics usually target cell proliferation, it could be possible that the effect on neurons is limited. Although the *RCN1* expression is relatively low in proliferative tissues, it is still present in many of them, suggesting that chemotherapeutics could have a stronger effect in these tissues. Nevertheless, these considerations are hypothetical and further research in this field needs to be done to assess target selectivity and tissue-specific toxicity.

Considering these aspects, we propose that *RCN1* can be used to complement existing biomarkers by offering additional prognostic insight and therapeutic relevance. While *VHL* mutations leading to elevated CAIX expression by stabilizing HIF present hallmarks of ccRCC [31],* RCN1* distinguishes itself by being involved in ER stress related and secretory pathways, suggesting a role apart from the hypoxia pathway or mutations in chromatin remodeling like *BAP1* or *PBRM1* [32]. Additionally, as *RCN1* is implicated in cancer biology beyond ccRCC, further investigation may offer novel therapeutic insight and accelerate drug development.

## Conclusion

This study confirmed that *RCN1* is highly expressed in ccRCC and associated with a shorter overall survival and poor clinico-pathological parameters, such as high grade, high stage and lymph node metastases. Furthermore, we were able to observe that reticulocalbin-1 is homogenously expressed in tumors and that a knockdown of *RCN1* can reduce the malign potential of renal tumor cells. Summarized, *RCN1* could be used not only as a prognostic biomarker but also in a therapeutic approach.

## Data Availability

The publicly available datasets analysed during the current study were obtained from The Cancer Genome Atlas (TCGA), available at the GDC data portal (Project ID: TCGA-KIRC) and from The Clinical Proteomic Tumor Analysis Consortium (CPTAC), available at [http://proteomics.cancer.gov]; (http://proteomics.cancer.gov) (Project: clear cell renal cell carcinoma). Further datasets used and analyzed during this study are available from the corresponding author on reasonable request.

## Abbreviations

ccRCC: Clear cell renal cell carcinoma
*RCN1*: Reticulocalbin-1
ER: Endoplasmic reticulum
FCS: Fetal calf serum

## References

[1] Feng X, Zhang L, Tu W, Cang S. Frequency, incidence and survival outcomes of clear cell renal cell carcinoma in the United States from 1973 to 2014: A SEER-based analysis. Medicine. 2019;98(31). 10.1097/MD.0000000000016684. 10.1097/MD.0000000000016684 PMC6708618 31374051

[2] Ljungberg B, Albiges L, Abu-Ghanem Y, Bensalah K, Dabestani S, Fernández-Pello S, et al. European Association of Urology Guidelines on Renal Cell Carcinoma: The 2019 Update. Eur Urology. 2019;75(5):799–810. 10.1016/j.eururo.2019.02.011. 10.1016/j.eururo.2019.02.011 30803729

[3] Motzer RJ, Jonasch E, Agarwal N, Alva A, Bagshaw H, Baine M, et al. NCCN Guidelines® Insights: Kidney Cancer, Version 2.2024. JNCCN. 2024;22(1):4–16. 10.6004/jnccn.2024.0008. 38394781 10.6004/jnccn.2024.0008

[4] Bedke J, Ghanem YA, Albiges L, Bonn S, Campi R, Capitanio U et al. Updated European Association of Urology Guidelines on the Use of Adjuvant Immune Checkpoint Inhibitors and Subsequent Therapy for Renal Cell Carcinoma. Eur Urology. 2025. 10.1016/j.eururo.2025.01.014. 10.1016/j.eururo.2025.01.014 39904712

[5] Heng DY, Xie W, Regan MM, Harshman LC, Bjarnason GA, Vaishampayan UN, et al. External validation and comparison with other models of the International Metastatic Renal-Cell Carcinoma Database Consortium prognostic model. A population-based study Lancet Oncol. 2013;14(2):141–8. 10.1016/S1470-2045(12)70559-4. 23312463 10.1016/S1470-2045(12)70559-4 PMC4144042

[6] Martini DJ, Liu Y, Shabto JM, Carthon BC, Hitron EE, Russler GA, et al. Novel Risk Scoring System for Patients with Metastatic Renal Cell Carcinoma Treated with Immune Checkpoint Inhibitors. Oncologist. 2020;25(3):e484–91. 10.1634/theoncologist.2019-0578. 10.1634/theoncologist.2019-0578 PMC7066702 32162798

[7] Suzuki H, Nagase S, Saito C, Takatsuka A, Nagata M, Honda K, et al. Raludotatug Deruxtecan, a CDH6-Targeting Antibody-Drug Conjugate with a DNA Topoisomerase I Inhibitor DXd, Is Efficacious in Human Ovarian and Kidney Cancer Models. Mol Cancer Ther. 2024;23(3):257–71. 10.1158/1535-7163.MCT-23-0287. 10.1158/1535-7163.MCT-23-0287 PMC10911705 38205802

[8] Yabe D, Taniwaki M, Nakamura T, Kanazawa N, Tashiro K, Honjo T. Human calumenin gene (CALU): cDNA isolation and chromosomal mapping to 7q32. Genomics. 1998;49(2):331–3. 10.1006/geno.1998.5245. 10.1006/geno.1998.5245 9598325

[9] Fukuda T, Oyamada H, Isshiki T, Maeda M, Kusakabe T, Hozumi A, et al. Distribution and variable expression of secretory pathway protein reticulocalbin in normal human organs and non-neoplastic pathological conditions. J Histochemistry Cytochemistry. 2007;55(4):335–45. 10.1369/jhc.6A6943.2006. 10.1369/jhc.6A6943.2006 17189526

[10] Suzuki N, Ban S, Itoh E, Chen S, Imai FL, Sawano Y, et al. Calcium-dependent structural changes in human reticulocalbin-1. J Biochem. 2014;155(5):281–93. 10.1093/jb/mvu003. 10.1093/jb/mvu003 24451493

[11] Chen X, Shao W, Huang H, Feng X, Yao S, Ke H. Overexpression of RCN1 correlates with poor prognosis and progression in non-small cell lung cancer. Hum Pathol. 2019;83:140–8. 10.1016/j.humpath.2018.08.014. 10.1016/j.humpath.2018.08.014 30172915

[12] Cooper CR, Graves B, Pruitt F, Chaib H, Lynch JE, Cox AK, et al. Novel surface expression of reticulocalbin 1 on bone endothelial cells and human prostate cancer cells is regulated by TNF-alpha. J Cell Biochem. 2008;104(6):2298–309. 10.1002/jcb.21785. 10.1002/jcb.21785 18561328

[13] Deng S, Pan Y, An N, Chen F, Chen H, Wang H, et al. Downregulation of RCN1 promotes pyroptosis in acute myeloid leukemia cells. Molecular Oncol. 2023;17(12):2584–602. 10.1002/1878-0261.13521. 10.1002/1878-0261.13521 PMC10701779 37746742

[14] Liu Z, Brattain MG, Appert H. Differential display of reticulocalbin in the highly invasive cell line, MDA-MB-435, versus the poorly invasive cell line, MCF-7. Biochem Biophys Res Commun. 1997;231(2):283–9. 10.1006/bbrc.1997.6083. 9070264 10.1006/bbrc.1997.6083

[15] Yin X, Wu Q, Hao Z, Chen L. Identification of novel prognostic targets in glioblastoma using bioinformatics analysis. Biomed Eng Online. 2022;21(1):26. 10.1186/s12938-022-00995-8. 35436915 10.1186/s12938-022-00995-8 PMC9014588

[16] Xu S, Xu Y, Chen L, Fang Q, Song S, Chen J, et al. RCN1 suppresses ER stress-induced apoptosis via calcium homeostasis and PERK-CHOP signaling. Oncogenesis. 2017;6(3). 10.1038/oncsis.2017.6. 10.1038/oncsis.2017.6 PMC5533947 28319095

[17] Huang ZH, Qiao J, Feng YY, Qiu MT, Cheng T, Wang J, et al. Reticulocalbin-1 knockdown increases the sensitivity of cells to Adriamycin in nasopharyngeal carcinoma and promotes endoplasmic reticulum stress-induced cell apoptosis. Cell cycle (Georgetown, Tex). 2020;19(13):1576–89. 10.1080/15384101.2020.1733750. 10.1080/15384101.2020.1733750 PMC7469451 32436770

[18] Wang JW, Ma L, Liang Y, Yang XJ, Wei S, Peng H, et al. *RCN1* induces sorafenib resistance and malignancy in hepatocellular carcinoma by activating c-MYC signaling via the IRE1α-XBP1s pathway. Cell Death Discovery. 2021;7(1):298. 10.1038/s41420-021-00696-6. 10.1038/s41420-021-00696-6 PMC8523720 34663798

[19] Fu H, Chen R, Wang Y, Xu Y, Xia C, Zhang B. Reticulocalbin 1 is required for proliferation and migration of non-small cell lung cancer cells regulated by osteoblast-conditioned medium. J Cell Mol Med. 2021;25(24):11198–211. 10.1111/jcmm.17040. 34747128 10.1111/jcmm.17040 PMC8650041

[20] Giribaldi G, Barbero G, Mandili G, Daniele L, Khadjavi A, Notarpietro A, et al. Proteomic identification of Reticulocalbin 1 as potential tumor marker in renal cell carcinoma. J Proteomics. 2013;91:385–92. 10.1016/j.jprot.2013.07.018. 23916412 10.1016/j.jprot.2013.07.018

[21] Qixin Y, Jing H, Jiang H, Xueyang L, Lu Y, Yuehua L. Transcriptome-based network analysis related to regulatory T cells infiltration identified *RCN1* as a potential biomarker for prognosis in clear cell renal cell carcinoma. BioData Mining. 2024;17(1):51. 10.1186/s13040-024-00404-x. 10.1186/s13040-024-00404-x PMC11566375 39543725

[22] Chandrashekar DS, Bashel B, Balasubramanya SA, Creighton CJ, Ponce-Rodriguez I, Chakravarthi BV, et al. UALCAN: A Portal for Facilitating Tumor Subgroup Gene Expression and Survival Analyses. Neoplasia (New York, NY). 2017;19(8):649–58. 10.1016/j.neo.2017.05.002. 10.1016/j.neo.2017.05.002 PMC5516091 28732212

[23] Chen F, Chandrashekar DS, Varambally S, Creighton CJ. Pan-cancer molecular subtypes revealed by mass-spectrometry-based proteomic characterization of more than 500 human cancers. Nature Commun. 2019a;10(1):5679. 10.1038/s41467-019-13528-0. 10.1038/s41467-019-13528-0 PMC6908580 31831737

[24] Chakiryan NH, Kimmel GJ, Kim Y, Hajiran A, Aydin AM, Zemp L, et al. Spatial clustering of CD68+ tumor associated macrophages with tumor cells is associated with worse overall survival in metastatic clear cell renal cell carcinoma. PloS One. 2021;16(4). 10.1371/journal.pone.0245415. 10.1371/journal.pone.0245415 PMC8059840 33882057

[25] Siegel RL, Miller KD, Fuchs HE, Jemal A. Cancer statistics. CA: Cancer J Clin. 2022;72(1):7–33. 10.3322/caac.21708. 35020204 10.3322/caac.21708

[26] Kurpińska A, Suraj J, Bonar E, Zakrzewska A, Stojak M, Sternak M, et al. Proteomic characterization of early lung response to breast cancer metastasis in mice. Exp Mol Pathol. 2019;107:129–40. 10.1016/j.yexmp.2019.02.001. 10.1016/j.yexmp.2019.02.001 30763573

[27] Nimmrich I, Erdmann S, Melchers U, Finke U, Hentsch S, Moyer MP, et al. Seven genes that are differentially transcribed in colorectal tumor cell lines. Cancer Letters. 2000;160(1):37–43. 10.1016/s0304-3835(00)00553-x. 11098082 10.1016/s0304-3835(00)00553-x

[28] Guo H, Shu J, Hu G, Liu B, Li J, Sun J, et al. Downregulation of *RCN1* inhibits esophageal squamous cell carcinoma progression and M2 macrophage polarization. PloS One. 2024;19(5):e0302780. 10.1371/journal.pone.0302780. 10.1371/journal.pone.0302780 PMC11075840 38713738

[29] Tumkur Sitaram R, Landström M, Roos G, Ljungberg B. Significance of PI3K signalling pathway in clear cell renal cell carcinoma in relation to VHL and HIF status. J Clin Pathol. 2021;74(4):216–22. 10.1136/jclinpath-2020-206693. 32467322 10.1136/jclinpath-2020-206693

[30] Liu H, Guo H, Wu Y, Hu Q, Hu G, He H, et al. *RCN1* deficiency inhibits oral squamous cell carcinoma progression and THP-1 macrophage M2 polarization. Sci Reports. 2023;13(1):21488. 10.1038/s41598-023-48801-2. 10.1038/s41598-023-48801-2 PMC10700561 38057406

[31] Wykoff CC, Beasley NJ, Watson PH, Turner KJ, Pastorek J, Sibtain A, et al. Hypoxia-inducible expression of tumor-associated carbonic anhydrases. Cancer Res. 2000;60(24):7075–83. 11156414

[32] Peña-Llopis S, Vega-Rubín-de-Celis S, Liao A, Leng N, Pavía-Jiménez A, Wang S, et al. BAP1 loss defines a new class of renal cell carcinoma. Nat Genet. 2012;44(7):751–9. 10.1038/ng.2323. 10.1038/ng.2323 PMC3788680 22683710

